# A Melanin-like Nanoenzyme for Acute Lung Injury Therapy via Suppressing Oxidative and Endoplasmic Reticulum Stress Response

**DOI:** 10.3390/pharmaceutics13111850

**Published:** 2021-11-03

**Authors:** Xue-Fang Lou, Chen Wang, Ju-Cong Zhang, Yong-Zhong Du, Xiao-Ling Xu

**Affiliations:** 1 School of Medicine, Zhejiang University City College, 51 Hu-Zhou Street, Hangzhou 310015, China; louxf@zucc.edu.cn; 2 Institute of Pharmaceutics, College of Pharmaceutical Sciences, Zhejiang University, Hangzhou 310058, China; 3170104036@zju.edu.cn (C.W.); 3170104034@zju.edu.cn (J.-C.Z.); 3 Shulan International Medical College, Zhejiang Shuren University, Hangzhou 310004, China

**Keywords:** melanin-like nanoenzyme, acute lung injury, endoplasmic reticulum stress, oxidative stress, 1,8-DHN polymerized nanoparticles

## Abstract

Nanoenzyme-mediated catalytic activity is emerging as a novel strategy for reactive oxygen species (ROS) scavenging in acute lung injury (ALI) treatment. However, one of the main hurdles for these metal-containing nanoenzymes is their potential toxicity and single therapeutic mechanism. Herein, we uncovered a melanin-like nanoparticles derived from the self-polymerization of 1,8-dihydroxynaphthalene (PDH nanoparticles), showing a significant anti-inflammation therapeutic effect on ALI mice. The prepared PDH nanoparticles rich in phenol groups could not only act as radical scavengers to alleviate oxidative stress but could also chelate calcium overload to suppress the endoplasmic reticulum stress response. As revealed by the therapeutic effect in vivo, PDH nanoparticles significantly prohibited neutrophil infiltration and the secretion of proinflammatory cytokines (TNF-α and IL-6), thus improving the inflammatory cascade in the ALI model. Above all, our work provides an effective anti-inflammatory nanoplatform by using the inherent capability of melanin-like nanoenzymes, proposing the potential application prospects of these melanin-like nanoparticles for acute inflammation-induced injury treatment.

## 1. Introduction

Acute lung injury (ALI) is a critical illness threatening public health [1,2,3] that is characterized serious inflammatory cell infiltration, pulmonary edema, and the formation of a hyaline membrane and interstitial fibrosis. Despite advances in pharmacotherapies and supportive care for ALI treatment, mortality and morbidity still remain unacceptably high (30–40%) [4,5]. The main reason for this could be attributed to the complex pathophysiological conditions based on the heterogeneity of etiological agents. Furthermore, the inflammatory cascade is quickly activated, resulting in rapid deterioration and possibly higher fatality. Thus, there is an urgent need to develop a more available therapeutic approach with multiple mechanisms for ALI treatment.

A variety of signaling pathways in pulmonary microvascular endothelial cells participate in the barrier permeability of ALI, including the increased intracellular calcium and reactive oxygen species (ROS) [6,7]. These two factors are regarded as key signaling events in the pathogenesis of ALI. Recently, researchers have pointed out that the expression of GRP78 and CHOP is upregulated in ALI models [8,9]. indicating that calcium overload-induced endoplasmic reticulum stress is involved in lung inflammation [10,11]. Furthermore, oxidative stress is often accompanied by abnormal increases in cytosolic calcium during endoplasmic reticulum stress in vivo [12,13,14]. It is presumed that both calcium overload and intracellular ROS generation can lead to mitochondrial dysfunction and then inflammatory cascade. Therefore, therapeutic interventions aiming to improve endoplasmic reticulum stress and oxidative stress may be warranted for the treatment of ALI.

The nanoenzyme is a type of nanomaterial with enzyme-like catalytic activity that has been developed in recent years, and it offers a new way of alleviating oxidative stress [15,16]. Since the first report of peroxidase-like activity of Fe_3_O_4_ nanoparticles in 2007 [17], significant progress has been made in the application of nanozymes for disease treatment. As most nanozymes are obtained by a metal or metal oxide, and their in vivo applications have been restricted due to long-term biosafety. Thus, researchers have made several efforts to develop metal-free nanozymes with catalytic activity. As a result, melanin-like nanoenzymes have begun receiving considerable research attention. Polydopamine, a type of melanin-like nanoenzyme derived from the self-polymerization of dopamine [18,19], was able to exhibit excellent ROS-scavenging ability due to its phenolic structure [20], offering a prospective effective treatment due to the amelioration of oxidative damage. Zhao et al. [21] developed bare PDA nanoparticles as an anti-inflammatory nano-drug for the treatment of ALI. The results demonstrated that the prepared PDA nanoparticles could significantly diminish ROS generation, decrease cytokine secretion, inhibit neutrophil infiltration, and improve lung tissue damage. Inspired by PDA, Zhou et al. [22] synthesized another melanin-like nanoparticle via the oxidative oligomerization of 1,8-dihydroxynaphthalene (1,8-DHN). The obtained nanoparticle could be taken up by neonatal human epidermal keratinocytes and could protect them from UV exposure by quenching ROS. However, to the best of our knowledge, the application of 1,8-DHN-based allomelanin nanoparticles as an anti-inflammatory agent to treat ALI has not yet been investigated. In addition, their ability to chelate calcium overload has not yet been explored.

In this work, bare 1,8-DHN-based allomelanin nanoparticles were prepared as anti-inflammatory nanoenzymes for ALI therapy. The preparation procedure for these melanin-like nanoenzymes was first optimized by adjusting the concentration of the oxidant, the stirring speed, and the water–oil ratio. Then, the obtained nanoparticles were utilized to investigate their capability to scavenge ROS and chelate calcium overload in vitro. The cellular internalization mechanism was further evaluated by adding different endocytosis inhibitors. The cytoplasmic levels of the ROS and free calcium were then imaged by a fluorescent probe. Afterwards, the biodistribution of indocyanine green-labeled allomelanin nanoparticles was conducted in ALI mice and normal mice. Finally, the pharmacodynamic evaluation was performed after the treatment of 1,8-DHN-based allomelanin nanoparticles for 48 h. Our design provides an effective anti-inflammatory nanoplatform by using the inherent capability of melanin-like nanoenzymes, suggesting the potential application prospect of these melanin-like nanoparticles as an anti-inflammatory nanomedicine.

## 2. Materials and Methods

### 2.1. Materials and Animals

The compounds 1,8-dihydroxynaphthalene (1,8-DHN), 3-(4,5-dimethylthiazol-2-yl)-2,5-diphenyltetrazolium bromide (MTT), and sodium periodate (NaIO_4_) were obtained from Sigma Chemical Co. (St. Louis, MO, USA). Indocyanine green (ICG) was purchased from Dalian Meilun Biotechnology Co., Ltd. (Dalian, China). Other chemicals applied in this research were of analytical or chromatographic grades.

BALB/c mice (6–8 weeks, male, 20 g) were provided by the Shanghai Silaike Laboratory Animal Limited Liability Company (Shanghai, China). They were given sufficient food and water during treatments. The animal experiments were conducted under the National Institutes of Health (NIH, Bethesda, MD, USA) guidelines for the care and use of laboratory animals in research. In addition, the surgical experiments were approved by the Committee for Animal Experiments of Zhejiang University (12033, 26 February 2018).

### 2.2. Synthesis of 1,8-DHN Polymerized Nanoparticles

We dissolved 20 mg of 1,8-DHN in 20 mL of acetonitrile (5%). Then, different volumes of NaIO_4_ (30 μL, 60 μL, 80 μL, 100 μL, and 120 μL) were added dropwise. After stirring at 400 rpm for 4 h, 1,8-DHN polymerized nanoparticles (PDH) were retrieved by centrifugation (11,000 rpm, 10 min) and were washed with water five times.

We dissolved 20 mg of 1,8-DHN in 20 mL of acetonitrile with different concentrations (5%, 10%, 20%, 50%, and 70%). Then, 100 μL of NaIO_4_ was added dropwise. After stirring at 400 rpm for 4 h, PDH nanoparticles were obtained by centrifugation (11,000 rpm, 10 min).

We dissolved 20 mg of 1,8-DHN in 20 mL of acetonitrile (5%). Then, 100 μL of NaIO_4_ was added dropwise. After stirring at different speeds (200 rpm, 400 rpm, 600 rpm, 800 rpm, and 1000 rpm) for 4 h, PDH nanoparticles were obtained by centrifugation (11,000 rpm, 10 min).

### 2.3. Characterization of PDH Nanoparticles

The size distribution and zeta potential of PDH nanoparticles on different days (day 1, 3, 8, 10, 15, and 18) were measured by dynamic light scattering (DLS, Zetasizer, Malvern Co., Malvern, UK) after their fabrication. The morphologies of the PDH nanoparticles were observed by transmission electron microscopy (TEM, JEOL JEM-1230, Tokyo, Japan).

The hemolysis assay was performed according to a previous study [23]. Red blood cells (RBCs) were harvested from rat blood after centrifugation (1500 rpm, 15 min) and were then diluted to a 2% suspension with saline. Afterwards, 0.5 mL of the RBC suspension was mixed with (1) 0.5 mL of saline as a negative control, (2) 0.5 mL of pure water as a positive control, and (3) 0.5 mL of PDH nanoparticles. In addition, 0.5 mL of PDH nanoparticles (1 mg/mL) mixed with saline served as a sample control. These samples were vortexed and maintained at room temperature for 3 h. In addition to the sample control, other samples were centrifuged at 8000 rpm for 5 min. The hemolysis images were recorded. The absorbance (*A*) of the supernatants at 541 nm was determined by a M5 full band multifunctional microplate reader (SynergyMx M5, Molecular Devices, San Francisco, CA, USA). The percent hemolysis of the RBCs was calculated as follows:

(1) $$Percent\;hemolysis(\%)=\frac{A_{sample}-A_{negative}}{A_{positive}-A_{negative}}\times 100\%$$

### 2.4. Ability to Scavenge ROS

Dichlorodihydrofluorescein (DCFH) can be rapidly oxidized to the strong fluorescent product 2′,7′-dichlorofluorescein (DCF) and is usually applied to react with ROS. Thus, DCFH (5 μM, prepared according to a previous study [24]) and PDH nanoparticles (1 mg/mL) were mixed. Then, the absorbances of PDH, DCFH, DCFH + H_2_O_2_, and DCFH + H_2_O_2_ + PDH under different wavelength were recorded using an M5 full-band multifunctional microplate reader.

### 2.5. Ability to Chelate Calcium Ions

PDH nanoparticles (1 mg/mL) were mixed with Ca^2+^-containing solutions (final concentration: 600 mg/mL) for different time points (10 min, 30 min, 120 min, and 480 min). Then, the nanoparticles chelating Ca^2+^ were obtained by centrifugation (11,000 rpm, 10 min), and the zeta potential was further measured. Meanwhile, the content of calcium ions was digested with aqua regia overnight and was then detected by inductively coupled plasma massspectrometry (ICP-MS, Thermo Fisher, Waltham, MA, USA).

### 2.6. Biocompatibility

Human umbilical vein endothelial cells (HUVECs, Shanghai Gefan Biotechnology Co., Ltd., P6, Shanghai, China) at the density of 1 × 10^4^ cells/well were seeded into 96-well plates and were incubated overnight at 37 °C in a 5% CO_2_ incubator. After 24 h, a series of PDH nanoparticle concentrations were added to each well. After another 48 h, MTT solution (5 mg/mL, 20 μL) was added. The culture continued for 4 h. Then, the supernatant was discarded, and 150 μL DMSO was added. The absorbance (*A*) at 570 nm was determined by an M5 full-band multifunctional microplate reader (SynergyMx M5, Molecular Devices, San Francisco, CA, USA). Cell viability was calculated according to Formula (2):

(2) $$Cell\;viability(\%)=\frac{A_{sample}}{A_{control}}\times 100\%$$

### 2.7. Cell Viability under Oxidative Conditions

HUVECs (1 × 10^4^ cells/well) were seeded into 96-well plates and were incubated overnight. After 24 h, cells were treated with H_2_O_2_ (the final concentration: 500 μM) and various concentrations of PDH nanoparticles. After another 48 h, cell viability was detected by MTT, as described in Section 2.6.

### 2.8. Annexin V-FITC and Propidium Iodide Staining

HUVECs (1 × 10^5^ cells/well) were seeded into 6-well plates and were incubated overnight. After 24 h, the cells were treated with H_2_O_2_ (the final concentration: 500 μM) and PDH nanoparticles (the final concentration: 50 μg/mL). After another 48 h, the HUVECs were collected and were resuspended in the binding buffer provided by the annexin V-FITC apoptosis detection kit (Beyotime Biotechnology Co. Ltd., Shanghai, China). Subsequently, annexin V-FITC (5 μL) and propidium iodide (10 μL) were added in turn. After incubation for 20 min (room temperature), the cells were examined using a flow cytometer (ACEA NovoCyteTM, ACEA Biosciences, San Diego, CA, USA).

### 2.9. Living Cells Staining

HUVECs (1 × 10^5^ cells/well) were seeded into 6-well plates. After attachment, the cells were treated with H_2_O_2_ (the final concentration: 500 μM) and PDH nanoparticles (the final concentration: 50 μg/mL) After another 48 h, the culture medium was removed. Cells were washed with PBS three times. Then, calcein AM (the final concentration: 10 μM)-containing medium was replaced, and the cells were incubated for 30 min. Then, the alive cells were immediately visualized under a fluorescence inverted microscope.

### 2.10. ROS Levels in HUVECs

The ROS levels in the HUVECs were detected according to a previous report [21]. HUVECs (1 × 10^5^ cells/well) were seeded into 6-well plates and were incubated overnight. Then, cells were treated with PDH nanoparticles (final concentration: 50 μg/mL) before adding H_2_O_2_ (the final concentration: 500 μM). After 30 minutes of exposure to H_2_O_2_, the culture medium was discarded and was replaced with 2,7-dichlorodihydrofluorescein diacetate (DCFH-DA, the final concentration: 10 μM)-containing medium. After incubation for 20 min, the cells were washed with PBS several times and were imaged by a fluorescence inverted microscope (Axio Observer A1, Zeiss, Oberkochen, Germany).

### 2.11. Calcium Content in HUVECs

HUVECs (1 × 10^5^ cells/well) were seeded into 6-well plates and incubated overnight. Then, the cells were treated with H_2_O_2_ (the final concentration: 500 μM) and PDH nanoparticles (the final concentration: 50 μg/mL). After another 48 h, the culture medium was removed. Cells was washed with PBS three times. Fluo-4 AM (the final concentration: 5 μM)-containing medium was added to the wells, and the cells were incubated for 30 min. Then, the cells were washed with PBS several times and were imaged by a fluorescence inverted microscope (Axio Observer A1, Zeiss, Oberkochen, Germany).

### 2.12. Western Blot

HUVECs (1 × 10^5^ cells/well) were seeded into 6-well plates and were incubated overnight. Then, cells were treated with H_2_O_2_ (the final concentration: 500 μM) and PDH nanoparticles (the final concentration: 50 μg/mL). After another 48 h, HUVECs were lysed with RIPA lysis buffer (Beyotime Biotechnology Co. Ltd., Shanghai, China). The protein concentration was determined using a BCA protein assay kit (Beyotime Biotechnology Co. Ltd., Shanghai, China). The same amount of protein for each group was added to the SDS-PAGE electrophoresis gel (8%). After electrophoresis, the membrane was probed overnight with GRP78 primary antibody (1:5000, abcam) at 4 °C. After the washing process, the membrane was incubated with secondary antibody (secondary ant rabbit IgG HRP; 1:1000) at room temperature for 2 h. Chemiluminescence agent (BeyoECL Plus, Beyotime Biotechnology Co. Ltd., Shanghai, China) was used for imaging and quantification in the Bio-Rad system (ChemiDoc Touch imaging system, Bio-Rad, Hercules, CA, USA).

### 2.13. Superoxide Dismutase (SOD) Activity

HUVECs (1 × 10^5^ cells/well) were seeded into 6-well plates and were incubated overnight. Then, cells were treated with H_2_O_2_ (final concentration: 500 μM) and PDH nanoparticles (final concentration: 50 μg/mL). After another 48 h, SOD activity in the HUVECs was evaluated using the total SOD activity test kit (Beyotime Biotechnology Co. Ltd., Shanghai, China).

### 2.14. Preparation of Indocyanine Green (ICG)-Labeled PDH Nanoparticles

PDH nanoparticles and ICG were weighed (1:1, *w*/*w*) and were then dissolved in ultrapure water. The ICG–PDH complexes were obtained after being stirred for 2 h and after centrifugation at 11,000 rpm for 10 min.

### 2.15. Cellular Uptake

HUVECs were seeded into 12-well plates and were classified into six groups: PBS + ICG-labeled PDH nanoparticle (50 μg/mL) for 2 h; H_2_O_2_ (500 μM) + ICG-labeled PDH nanoparticles (50 μg/mL) for 2 h; PBS + ICG-labeled PDH nanoparticles (50 μg/mL) for 4 h; H_2_O_2_ (500 μM) + ICG-labeled PDH nanoparticles (50 μg/mL) for 4 h; PBS + ICG-labeled PDH nanoparticle (50 μg/mL) for 6 h; and H_2_O_2_ (500 μM) + ICG-labeled PDH nanoparticles (50 μg/mL) for 6 h. After the washing process, the cells were collected and detected by flow cytometry (ACEA NovoCyteTM, ACEA Biosciences, San Diego, CA, USA, FL channel: APC-A750, numeber of events: 100 μL). HUVECs without any treatment were used as a control.

### 2.16. Mechanism of Cellular Uptake

Cells were seeded at 2 × 10^4^ cells per well in a 48-well plate. The cells were pretreated with a clathrin-mediated uptake inhibitor (Chlorpromazine, 25 μM), caveolin-mediated uptake inhibitor (indomethacin, 50 μM), macropinocytosis inhibitor (amiloride, 50 μM), and lipid raft-mediated uptake inhibitor (M-β-CD, 10 μg/mL) for 30 min. The H_2_O_2_ and ICG–PDH complexes were added, and cultures were incubated for another 4 h. After the washing process, the cells were collected and detected by flow cytometry (ACEA NovoCyteTM, ACEA Biosciences, San Diego, CA, USA; FL channel: APC-A750, numeber of events: 10,000 cells).

### 2.17. Lysosome-Tracking Experiments

HUVECs were treated with H_2_O_2_ and ICG-labeled PDH nanoparticles for 4 h. Lysosomes were dyed with LysoTracker (Beyotime Biotechnology Co. Ltd., Shanghai, China) at a concentration of 50 nM for 30 min. After being washed three times with PBS, the fluorescence in the cells was monitored using a laser confocal microscope (Olympus BX61, Olympus Ltd., Tokyo, Japan).

### 2.18. Biodistribution

The ALI model was established via the intratracheal instillation of a lipopolysaccharide solution (LPS, 2 mg/kg) [25,26]. Once completed, the mice were quickly erected to promote the distribution of the instilled solution into the lung. The ALI model was established after stimulation for 6 h. Mice were randomly distributed into two groups: the control (healthy mice) and the ALI model.

The control and ALI mice were intravenously injected with ICG–PDH nanoparticles at a dose of 10 mg/kg. After 2 h, 12 h, 24 h, and 48 h, mice were euthanized, and their organs were collected shortly after. The Maestro in vivo imaging system (Caliper, Hopkington, MA, USA) was used to measure the fluorescence signals in the mouse organs. The parameters were set at: filter = ICG 790 nm; exposure time = 1440 ms.

### 2.19. Acute Lung Injury Treatment

Mice were randomly distributed into four groups: (1) healthy mice (*n* = 6); (2) ALI mice treated with PBS (*n* = 6); (3) ALI mice treated with methylprednisolone hemisuccinate (*n* = 6); and (4) ALI mice treated with PDH nanoparticles (*n* = 6). Methylprednisolone hemisuccinate is the primary drug in clinical ALI treatment, so it was used as the positive control in this study.

A total of 48 h after administration, the mice were sacrificed, and their lungs were collected and weighed.

The organs (heart, liver, spleen, lung, and kidneys) were collected and were fixed with 4% paraformaldehyde for 48 h. Then, the embedded organs were cut with a microtome to prepare 5-μm-thick sections and were dried for HE staining.

After sacrifice, the mice were fixed in the supine position. Bronchoalveolar lavage fluid (BALF) was obtained. The collected BALF was quickly centrifuged at 1300 rpm for 10 min, and the supernatant was harvested and stored at −80 °C until use.

The precipitate was then lysed using the red blood cell lysis buffer (Beyotime Biotechnology Co. Ltd., Shanghai, China) for 1 min. Thereafter, 7 mL PBS was added to stop the lysis process. Then, the mixture was centrifuged at 1300 rpm for 10 min. The obtained precipitate was further mixed with 500 μL of PBS to form a cell suspension, and 50 μL of this cell suspension was used to analyze the total cell counts using a hemocytometer. The remaining cells were treated with PE-labeled rat anti-mouse Ly-6G/Ly-6C monoclonal antibody (Elabscience, Wuhan, China, 50 tests) and FITC-labeled rat anti-mouse CD11b monoclonal antibody (Elabscience, 50 tests) at 4 °C for 20 min. Then, the cell samples were washed with PBS and were centrifuged at 1300 rpm for 10 min. The precipitate was resuspended and was evaluated by flow cytometry (FL channel: FITC, PE; number of events: 10,000 cells).

The collected lungs were cleaned and lysed. After centrifugation (13,000 rpm, 15 min), the supernatant was harvested. The levels of TNF-α and IL-6 in the supernatant were further measured by ELISA kits (Boster Biological Technology Co. Ltd., Wuhan, China).

### 2.20. Statistical Analysis

Comparative analysis of the differences between groups was calculated by one-way analysis of variance or t test with Prism 7.0 (95% confidence interval, GraphPad Software, San Diego, CA, USA). A significant difference was set at *** *p* < 0.001, ** *p* < 0.01, and * *p* < 0.05. Values are displayed in the form of mean ± standard deviation.

## 3. Results

### 3.1. Characterization of PDH Nanoparticles

In the process of preparing PDH nanoparticles, the oxidant content, water–oil ratio, and stirring speed are the key conditions that must be considered to control the particle size of nanoparticles. As displayed in Table 1, with the increase in the NaIO_4_ content, the particle size increased first and then decreased over time. In turn, the polydispersity index (PDI) of these prepared nanoparticles decreased gradually and then increased. A previous report [22,27] demonstrated that the 1,8-DHN monomer was first oxidized, forming several 1,8-DHN free radicals. Subsequently, a suitable dimer was obtained through the coupling of these free radicals, including three main types of dimers (2–2′, 4–4′ and 2–4′ dimer). These dimers further oxidized, oligomerized, and self-assembled through molecular hydrogen bonds to form spherical nanoparticles. In addition, a higher proportion of NaIO_4_ can generate higher molecular weight oligomers, which may affect the size distribution and PDI of nanoparticles. Regarding the stirring speed, it had a minimal effect on the particle size (at around 115 nm), while the nanoparticles prepared at medium rotational speed had a lower PDI. In contrast to stirring speed, the water–oil ratio had a significant influence on the particle size of the nanoparticles, as reflected by the smaller particle size with the higher water–oil ratio. The solubility of the 1,8-DHN monomer in different water–oil ratios may induce different oxidation levels since the concentration of monomers in the reaction is different. Moreover, the results of the PDI demonstrate that the dispersion became larger with an increase in the water–oil ratio. Accordingly, a better condition to prepare more stable PDH nanoparticles could be set at: 5% NaIO_4_, 400 rpm, and 5% acetonitrile. The particle size and PDI of the obtained nanoparticles were 126.17 ± 10.21 nm and 0.039 ± 0.049, respectively.

**Figure 1:**
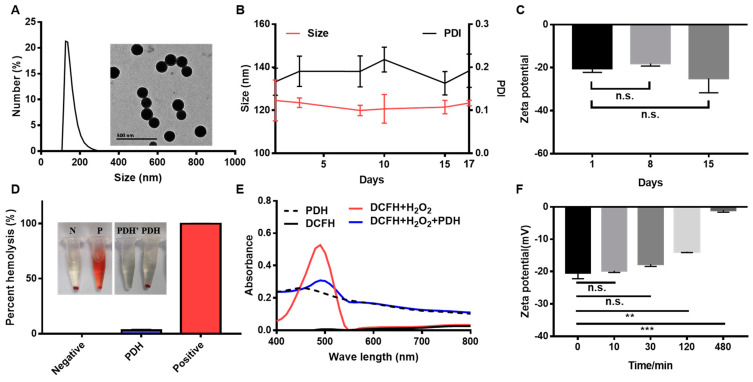
Characterization of PDH nanoparticles. (**A**) The size distribution and TEM images (inserted) of PDH nanoparticles. (**B**) Changes in nanoparticle size and polydispersity index after storage for 1, 3, 8, 10, 15, and 17 days. (**C**) Changes in zeta potential after storage for 1, 8, and 15 days. (**D**) The hemolysis rate and images (inserted) of red blood cells after incubation with different nanoparticles. N: negative control, P: positive control, PDH’: PDH sample mixed with saline, PDH: PDH sample mixed with red blood cells. (**E**) The absorbance of PDH, DCFH, DCFH + H_2_O_2_, and DCFH + H_2_O_2_ + PDH under different wavelengths. (**F**) Changes in zeta potential of PDH nanoparticles after incubation with calcium ion-containing solution (calcium chloride aqueous solution). (Results are presented as mean ± SD, n.s. is non-significant, ** *p* < 0.01, *** *p* < 0.001, *n* = 3).

Figure 1 presents the characterization of the obtained PDH nanoparticles. As displayed, the PDH nanoparticles are shaped as spheres with a diameter of about 125 nm (Figure 1A). After storage for 1, 3, 8, 10, 15, and 17 days (room temperature), these nanoparticles did not significantly change over time in regard to their particle size and PDI (Figure 1B). Meanwhile, the zeta potential was also measured. It should be noted that the PDH nanoparticles showed non-significant changes in terms of zeta potential on days 1, 8, and 15 (Figure 1C). In the in vitro hemolysis assay, the hemolysis rate was 3.11 ± 0.77% for PDH nanoparticles (Figure 1D). Considering that the hemolysis rate of the biomaterials is limited to 5%, PDH-based micelles showed good blood compatibility. Moreover, dichlorodihydrofluorescein diacetate (DCFH) was used as a probe to detect the ROS scavenging activity of PDH nanoparticles toward H_2_O_2_. In the presence of H_2_O_2_, DCFH was oxidized and showed an absorbance peak at 488 nm. Interestingly, it could be observed that the addition of PDH rapidly reduced the absorbance peak at 488 nm in the solution (Figure 1E). Following these observations, the ability to chelate calcium ions was further analyzed. With the incubation time extended, the zeta potential of PDH nanoparticles decreased (Figure 1G), implying an increasing calcium chelating capability. ICP-MS was employed to detect the calcium ion concentration in the nanoparticles. Notably, 45.66 ± 5.69% of the calcium ions could be chelated in 4 h.

### 3.2. In Vitro Antioxidant Effect of PDH Nanoparticles

**Figure 2:**
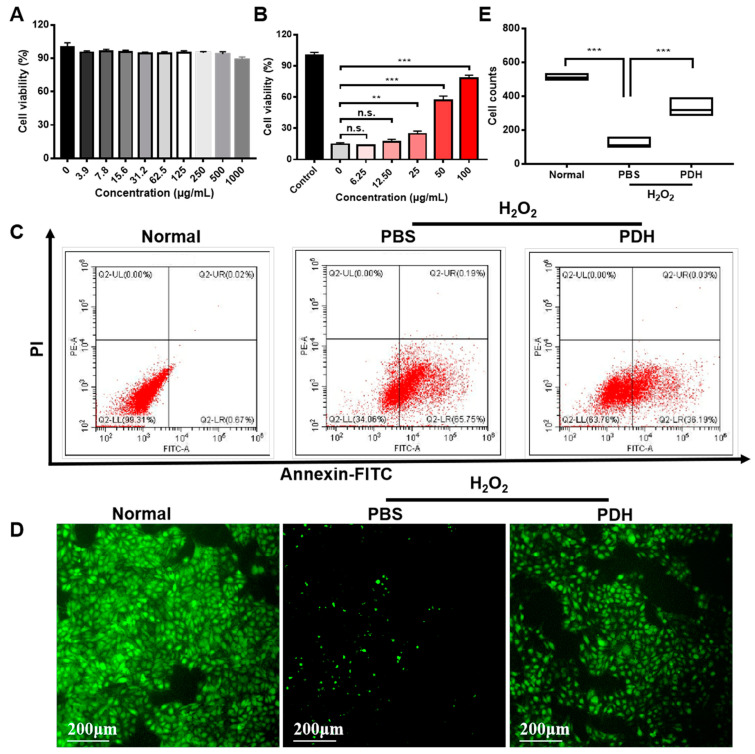
In vitro therapeutic effect of PDH nanoparticles. (**A**) The cell viability of PDH nanoparticles in normal HUVECs detected by MTT assay. (**B**) The cell viability after treatment with different concentrations of PDH nanoparticles detected by MTT assay. (**C**) The annexin-FITC and PI staining of HUVECs after different treatments evaluated by flow cytometry. The first quadrant indicates living cells. The second quadrant indicates early apoptotic cells. The third quadrant represents late apoptotic cells. The fourth quadrant shows the necrotic cells. (**D**) Living cells (labeled with calcein AM) after different treatment with PDH. (**E**) Cell count in Figure 2D measured by image J. (n.s. is non-significant, ** *p* < 0.01, *** *p* < 0.001, *n* = 3).

Figure 2 shows the in vitro antioxidant effect of PDH nanoparticles. First, the MTT assay was applied to test the number of metabolic cells in the culture. The effect of PDH nanoparticles on cytotoxicity was investigated in normal HUVECs. The results demonstrated that even when the PDH concentration was as high as 1000 μg/mL, cell viability was still more than 90% (Figure 2A), indicating that the melanin-like nanoparticles had good biocompatibility. Then, H_2_O_2_ was used to stimulate HUVECs to establish an oxidative stress model. The effect of PDH nanoparticles on cell viability was evaluated under oxidative stress conditions. As shown, the cell viability decreased from 100% to 14.76% in the presence of H_2_O_2_ (Figure 2B). After PDH treatment, the cell viability gradually increased along with the nanoparticle concentrations. At the concentration of 100 μg/mL, the cell viability reached 78.01%. Although the MTT test could provide information on the number of metabolic cells, a limitation was that an independent increase in the MTT reduction might occur in the presence of an oxidative stress or when the cellular oxidoreductases responsible for MTT reduction are affected by nanoparticles.

Since the early redistribution of phosphatidylserine is a general feature of apoptosis and since annexin-FITC could specifically combine with phosphatidylserine, annexin-FITC and propidium iodide (PI) staining was further used to monitor the number of apoptotic cells. The anti-apoptotic effect of PDH nanoparticles was detected by annexin-FITC and PI staining via flow cytometry. As displayed, the proportion of apoptotic cells significantly increased (from 0.67% to 65.75%, Figure 2C). After PDH treatment, the proportion reduced to 36.19% (Figure 2C).

Calcein acetoxymethyl ester (calcein AM) is a fluorescent indicator that can provide information on the oxidative status of cells. It is hydrolyzed by living cells to produce calcein, which can emit strong green fluorescence. This allowed the effect of the PDH nanoparticles on the number of living cells to be investigated under oxidative stress conditions. As displayed, there are few fluorescent cells in the visual field of the PBS-treated group, indicating few living cells (Figure 2D). In contrast, the PDH-treated group demonstrated a significant increase in fluorescent cells, implying that more HUVECs survived after PDH treatment under H_2_O_2_-induced oxidative stress compared to the PBS group (123 versus 332, Figure 2E). During the probe loading process, cells should be washed completely to avoid the influence of serum, oxidative stress, and nanoparticles, but as a result of this, newborn cells or cells without attachment may be removed.

**Figure 3:**
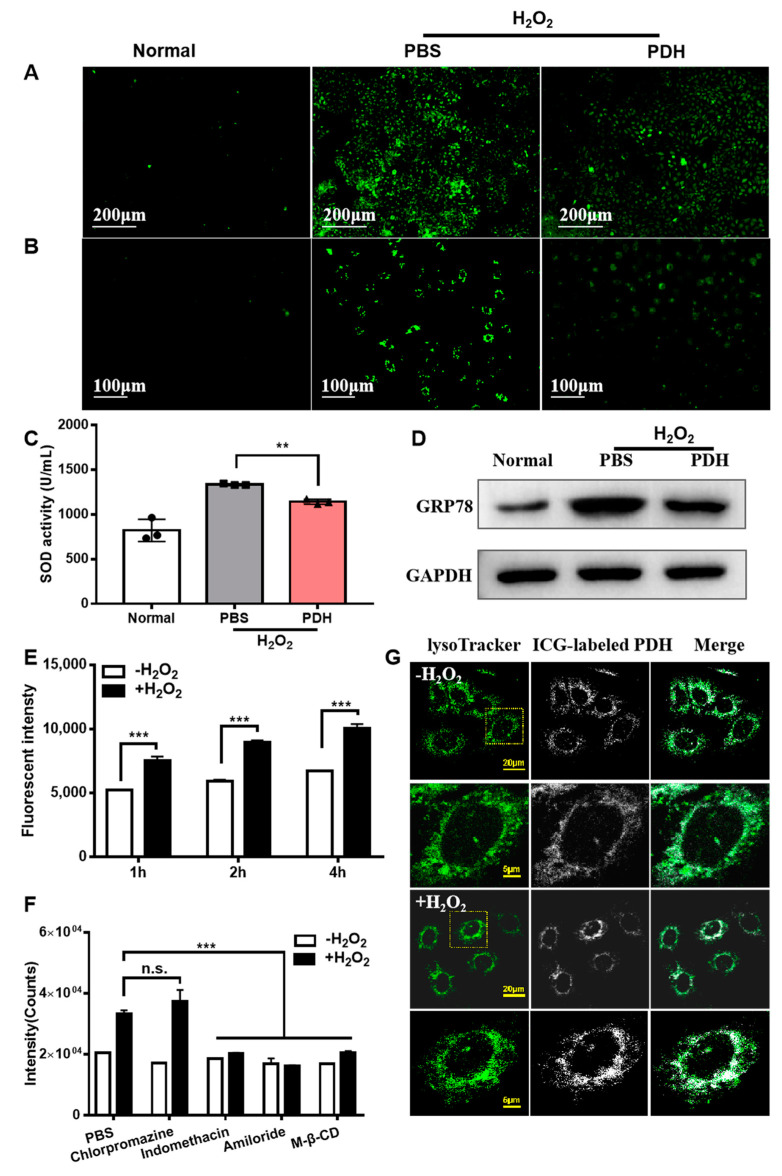
In vitro therapeutic mechanism of PDH nanoparticles (50 μg/mL). (**A**) The ROS levels in cells after different treatments using the DCFH-DA probe. (**B**) The concentrations of free calcium ions in cells after different treatments detected with Fluo-4 AM probe. (**C**) The SOD activity in cells after different treatments was detected by total SOD assay kit with WST-8. Three circles in the figure mean three samples detected in the normal group. Three squares indicate three samples investigated in the PBS group. And three triangles represent three samples detected in the PDH group. (**D**) The expression levels of GRP78 in cells after different treatments were analyzed by Western blot. (**E**) The fluorescent intensities in cells treated with ICG-labeled PDH nanoparticles in the presence or absence of H_2_O_2_ for 1 h, 2 h, and 4 h were measured by flow cytometry. (**F**) The fluorescent intensity at 4 h in cells pretreated with different internalization inhibitors was measured by flow cytometry. (**G**) The co-localization of the lysosome tracker and ICG-labeled PDH nanoparticles at 4 h. (n.s. is non-significant, ** *p* < 0.01, *** *p* < 0.001, *n* = 3).

Figure 3 exhibits the antioxidant mechanism of the PDH nanoparticles. The compound 2,7-Dichlorodihydrofluorescein diacetate (DCFH-DA) is a general fluorescent probe of oxidative stress. It can be hydrolyzed by cellular esterase to produce 2′,7′-dichlorodihydrofluorescein (DCFH), and it can then be rapidly oxidized to produce a strong fluorescent product 2′,7′-dichlorofluorescein (DCF). Accordingly, the effect of PDH nanoparticles (50 μg/mL) on intracellular ROS levels was monitored. As shown in Figure 3A, a large quantity of fluorescent cells was observed in the PBS-treated group, revealing overproduced cellular ROS. PDH nanoparticles significantly reduced the fluorescent counts compared to the PBS group, implying that more ROS were scavenged. Considering that ROS had a short lifespan and were not easy to detect, the cells were first exposed to the PDH nanoparticles with the aim of internalization into cells. Reduced fluorescent ROS signals implied that the PDH nanoparticles effectively scavenged the intracellular ROS, hence protecting the endothelial cells against enhanced oxidative stress.

The intracellular ROS levels was associated with the SOD activity. A total SOD assay kit was used to detect the enzyme activity. Thus, the effect of PDH treatment on SOD activity could be evaluated. As evidenced, a reduced SOD activity could be observed in the PDH group (1334.24 U/mL) when compared to the PBS group (1141.77 U/mL, Figure 3C).

Since mass calcium ion swimming to the cytoplasm may result in endoplasmic reticulum stress, the cellular concentration of free calcium ions was detected. The Fluo-4 AM is a fluorescent indicator of free calcium ions. Its fluorescence is very weak and does change when the calcium ion concentration increases. Once the cells enter, Fluo-4 AM can be hydrolyzed to form Fluo-4, which can be combined with calcium ions and can produce strong fluorescence. Hence, the effect of the PDH nanoparticles on the cellular concentration of calcium ions was investigated. Clearly, H_2_O_2_ stimulation induced strong green fluorescence, indicating abundant calcium ion release into cytoplasm. Interestingly, PDH treatment induced a significant decrease in the fluorescent signals related to calcium in comparison to the PBS group (Figure 3B). Since the PDH nanoparticles preferred to locate in the cytoplasm, this led to a strong chelating ability on the calcium ion overload.

Calcium ion overload was essentially involved in endoplasmic reticulum stress. GRP78 is a general indicator of endoplasmic reticulum stress. Thus, the effect of the PDH nanoparticles on the inhibition of the endoplasmic reticulum stress response was assessed via the expression of GRP78. As evidenced, the expression level of GRP78 was significantly upgraded after H_2_O_2_ stimulation (Figure 3D). In contrast, PDH treatment decreased the GRP78 expression level.

To determine the mechanism of the PDH nanoparticles in suppressing the oxidative and endoplasmic reticulum stress response, an ICG-labeled PDH nanoparticle was prepared first, and its cellular uptake was further conducted. Stronger fluorescence intensity indicates the high internalization of ICG-labeled PDH nanoparticles into cells. It can be observed that the cellular fluorescence intensity increased with the extension of the incubation time (Figure 3E). Moreover, the further internalization of the PDH nanoparticles into the cells was detected in the presence of H_2_O_2_. The involvement of H_2_O_2_ causes cell membrane permeability to be enhanced [28]; thus, the ICG-labeled PDH can easily infiltrate cells.

To investigate how PDH nanoparticles enter cells, different inhibitors of the endocytic pathways were used. The results showed that caveolin-mediated endocytosis, micropinocytosis, and lipid raft-mediated uptake made an important contribution to the internalization of PDH nanoparticles because the uptake processes were inhibited with various inhibitors, such as indomethacin (inhibitor of caveolin-mediated endocytosis), amiloride (inhibitor of macrocytosis), and methyl-β-cyclodextrin (inhibitor of lipid raft-mediated endocytosis). The cellular fluorescent signals reduced dramatically compared to the control group (Figure 3F). Theoretically, caveolin-mediated endocytosis could form caveosomes to escape lysosomal capture, enabling the PDH nanoparticles to cross the internal organelle barrier and eventually reach the cytoplasm to chelate calcium ions and scavenge excessive ROS.

To further detect the ICG-labeled PDH nanoparticles located in the cytoplasm, the lysosome was tracked by a lysotracker. Green represents the lysosome, while white indicates ICG-labeled PDH nanoparticles. Thus, based on the co-location/separation of green and white, the distribution of ICG-labeled PDH nanoparticles could be demonstrated. It was found that a large number of PDH nanoparticles were co-localized with the lysosome, indicating more accumulation in the lysosome before H_2_O_2_ stimulation. On the contrary, PDH nanoparticles revealed less co-localization with the lysosome, suggesting that more PDH nanoparticles entered into the cytoplasm. Lysosomes are organelles with many kinds of hydrolases, which can degrade nanoparticles. Therefore, the distribution of PDH–ICG complexes in the cells was an essential step for functioning. Having more PDH nanoparticles distributed in the cytoplasm contributed to a better effect on scavenging ROS and chelating Ca^2+^.

### 3.3. In Vivo Antioxidant Effect of PDH Nanoparticles

**Figure 4:**
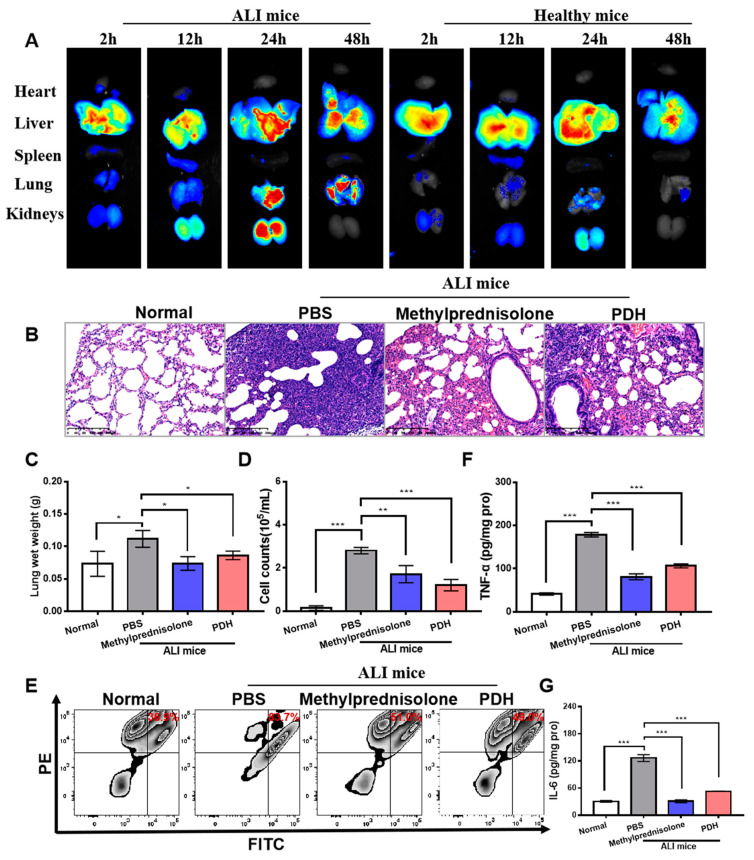
In vivo therapeutic effect of PDH nanoparticles on ALI-induced mice. (**A**) Biodistribution of PDH/ICG in vivo. The fluorescence images of harvested organs from mice treated with LPS or not at 2 h, 12 h, 24 h and 48 h. (**B**) Morphologic alterations in lungs after different treatments were identified by hematoxylin and eosin staining. Scale bar = 200 µm. (**C**) The lung wet weight. (**D**) The total cell counts in the bronchoalveolar lavage fluid detected by flow cytometry. (**E**) The neutrophil counts in the bronchoalveolar lavage fluid measured by flow cytometry. (**F**) The level of TNF-α in lung tissues detected by TNF-α ELISA kit. (**G**) The level of IL-6 in lung tissues detected by IL-6 ELISA kit. (* *p* < 0.05, ** *p* < 0.01, *** *p* < 0.001, *n* = 3).

To assess the biodistribution of PDH nanoparticles in vivo, ALI-induced or healthy mice were injected intravenously with ICG-labeled PDH nanoparticles. After administration for 2 h, 12 h, 24 h, and 48 h, the organs were harvested and imaged. As displayed in Figure 4A, the fluorescence signals increased in time and peaked at about 24 h. In healthy mice, a strong fluorescence signal could be observed in the liver rather than in the kidneys. However, in ALI mice, the fluorescent intensity in the kidneys increased, followed by a decreased fluorescence signal in the liver. In addition, this fluorescent signal preserved at 48 h in the ALI mice, while in normal mice, the fluorescence quickly decreased.

The therapeutic efficacy of PDH nanoparticles was assessed in the ALI murine model. As shown in Figure 4B, the lung of PBS-treated mouse showed significant pathological changes, as reflected by alveolar wall thickening, even alveolar disappearance, and infiltration of various inflammatory cells. After treatment, histological improvement could be noticed (alveolar structure and inflammatory cell infiltration). Compared to the PBS group, PDH-treated mice showed significantly improved inflammatory cell infiltration and alveolar wall thickening. Since inflammatory cell infiltration may influence organ weight, the lung wet weight was analyzed. As shown in Figure 4C, the PBS-treated group had a higher lung wet weight than the normal group (0.112 g versus 0.073 g). Nevertheless, the lung wet weight significantly decreased in the methylprednisolone (0.074 g) and PDH (0.086 g) groups. This higher lung weight may be associated with enhanced inflammatory cell infiltration. To validate these findings, the total cell number in the pulmonary interstitial fluid was measured. As shown in Figure 4D, the PBS group displayed a sharp increase in the total cell counts after LPS challenge at 48 h (280,687/mL versus 14,617/mL). When the mice were injected with PDH, the total cell counts showed a significant decrease (120,627/mL). Among all of the inflammatory cells, neutrophil cells exerted a crucial role in the inflammatory cascade in the process of ALI development. Thus, pulmonary inflammatory cell infiltration was further monitored via the specific identification of CD11b and Ly6G (FITC-CD11b+ and PE-Ly6G+). As displayed, the neutrophil cell number increased from 36.90% to 83.70% in the bronchoalveolar lavage fluid after LPS stimulation (Figure 4E). Nevertheless, both the methylprednisolone (51.00%) and PDH group (49.00%) inhibited neutrophil cell infiltration. Pro-inflammatory cytokines (such as TNF-α and IL-6) are another indicator that can be used to evaluate inflammatory cascades in the development of ALI. Therefore, the levels of TNF-α and IL-6 in the lung tissues were further detected by ELISA kits. The results showed that the PBS-treated mice secreted more TNF-α and IL-6 in the lungs compared to the normal group (TNF-α: 179 pg/mg pro versus 41 pg/mg pro, IL-6: 31 pg/mg pro versus 126 pg/mg pro). Notably, the PDH group significantly inhibited the secretion of inflammatory cytokines.

### 3.4. Biosafety

**Figure 5:**
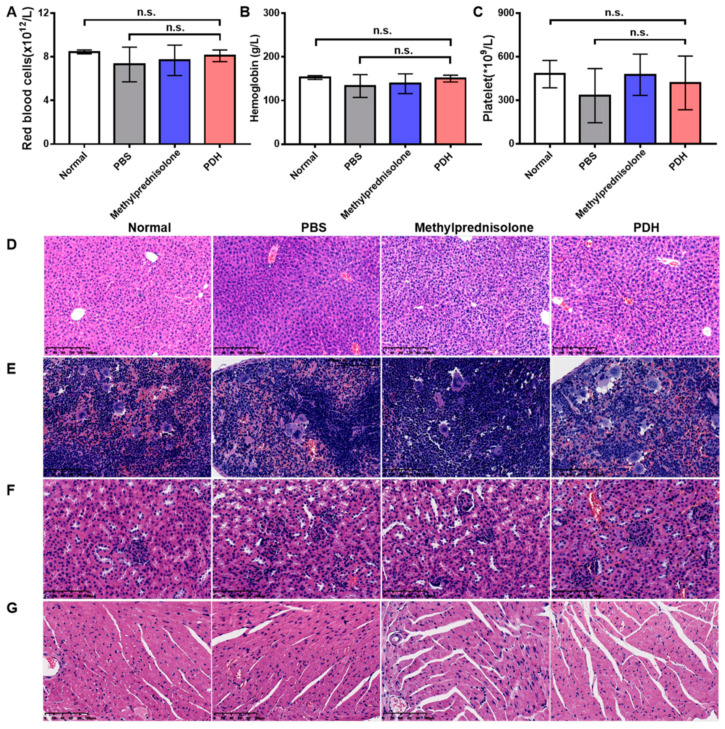
The biosafety of PDH nanoparticles in ALI mice. The changes in red blood cells (**A**), hemoglobin (**B**), and platelets (**C**) detected by automatic blood analyzer (n.s. is non-significant, *n* = 6). Representative images of liver ((**D**), scale bar = 200 μm), spleen ((**E**), scale bar = 100 μm), kidney ((**F**), scale bar = 100 μm), and heart ((**G**), scale bar = 100 μm) identified with hematoxylin and eosin staining.

The adverse effect of PDH nanoparticles was evaluated by analyzing the counts of red blood cells, hemoglobin, and platelets. The results showed that PDH nanoparticles induced non-significant changes in the level of red blood cells (Figure 5A), hemoglobin (Figure 5B), and platelets (Figure 5C) compared to the untreated ALI mice or untreated normal mice. Moreover, H&E staining revealed that PDH did not induce visible damage to the liver (Figure 5D), spleen (Figure 5E), kidneys (Figure 5F), or heart (Figure 5G), as reflected by the regular structure of myocardial fibers, spleen parenchyma, hepatic lobules, and renal tubules compared to the normal group. These results further confirm the biosafety of the prepared nanoparticles.

## 4. Discussion and Conclusions

Endoplasmic reticulum stress caused by calcium overload and excess ROS are considered to be two important pathological mechanisms of inflammatory response syndrome in ALI [11,29,30]. Melanin-like nanoenzymes are a series of macromolecules that contain phenolic hydroxyl and amino and imino acids in its structural units, which have been shown to have the functions of light protection [22], free radical capture [20], and photothermal conversion [31].

In the current study, 1,8-DHN were used as monomers to prepare melanin-like nanoenzymes. First, the preparation process was optimized by adjusting the concentration of oxidant, the stirring speed, and the water–oil ratio. After optimization, a nanoenzyme with a particle size of 126 nm was obtained. The prepared melanin-like nanoenzymes could be rapidly distributed to the injured lung at 2 h after LPS challenge, and the accumulation reached its maximum at 24 h. Then, they entered the cytoplasm in large quantities through caveolin-mediated endocytosis. Once the nanoparticles were taken up by the cells, the nanoparticles chelated the overloaded calcium ions, contributing to an improvement in endoplasmic reticulum stress. At the same time, the excess ROS in the cytoplasm were removed, improving the ability of the cells to deal with oxidative stress and to inhibit the apoptosis of the injured endothelial cells. The in vivo antioxidant experiments at 48 h demonstrated that PDH could effectively decrease the infiltration of neutrophils in the ALI mice lung tissue and could significantly reduce the wet weight of the lung tissue and the secretion levels of inflammatory factors (TNF-α and IL-6). All of these improvements inhibited the pulmonary inflammatory response, thus preventing further injury.

The major strengths of the PDH nanoparticles for ALI can be summarized in four aspects: (i) PDH nanoparticles with a diameter of 126 nm exert functions that intrinsically inhibit the oxidative and endoplasmic reticulum stress response; (ii) the therapeutic mechanism is diverse, including scavenging ROS and chelating calcium ions; (iii) PHD nanoparticles demonstrate the potential to be nanocarriers, which suggests that they could be nanodrug delivery systems, providing an improved therapeutic effect; and (iv) the biosafety level is appropriate during treatment.

PDH nanoparticles have demonstrated many benefits and potential in ALI therapy. However, their use remains in the early stage and is still far from entering the clinical trial phase. We have yet to find solutions to considerable problems that challenge the therapeutic effect of these melanin-like nanoparticles:

(1) Biodistribution: Although a large quantity of PDH was accumulated in the injured lung and no obvious pathological changes were observed during treatment, the lung targeting efficiency remained unsatisfactory. An improvement in nanoparticles is necessary, and this can be achieved, for example, by choosing an appropriate particle size or by engineering the nanoparticle surface. A previous study [32] demonstrated that particle size significantly influenced the fate of nanoparticles, leading to different biodistribution and cellular uptake. However, there remains controversy regarding the notion that nanoparticles with a specific particle size may achieve maximum lung accumulation [33]. Hence, it is very important to investigate the lung aggregation of PDH nanoparticles with different particle sizes in inflammatory lungs. Moreover, some targeting moieties or membranes can be applied onto the surface of nanoparticles to circumvent the biologic obstacles, resulting in improved accumulation in lesions.

(2) Animal model: Previous experiments have showed that the tested drug always results in failure in clinical trials, although a good therapeutic effect in animal models has been demonstrated [33]. This indicates that the development of an accurate ALI model remains a challenge. Therefore, it is necessary to explore the complicated pathogenesis of ALI, followed by the establishment of an accurate ALI animal model for ALI treatment.

(3) Therapeutic mechanism: ALI is associated with hypoxia, bacterial/virus infections, endoplasmic reticulum stress, oxidative stress, inflammatory response, and so on. Moreover, many target cells are related to ALI, including neutrophils, macrophages, and endothelial cells. Due to these complex pathophysiological conditions induced by the heterogeneity of etiological agents, a single drug or pathway for ALI therapy is not sufficient. In contrast, targeting different types of damaged cells or multiple pathways in the same cells simultaneously will significantly improve the therapeutic effect of ALI.

In summary, melanin-like nanoenzymes or melanin-like nanoenzyme-based drug delivery systems show great potential for clinical therapeutic perspectives. They represent the first drug delivery system that can both chelate Ca^2+^ overload and that can eliminate excess ROS, providing a novel strategy for the development of antioxidant nanomedicines. An appropriate particle size, engineered surface, accurate animal model, and coordinated therapy of multiple targets/pathways can improve the therapeutic effect, which may subsequently allow for gaps in both basic and clinical research to be filled. Considering that patients suffering from COVID-19 also show some symptoms similar to those of ALI, melanin-like nanoenzyme-relevant research may facilitate the identification of potential therapy options for COVID-19.

## Figures and Tables

**Table 1:** Size of polydispersity index of PDH nanoparticles.

Parameters		Size (nm)	Polydispersity Index (PDI)
NaIO_4_ (%, *w*/*v*)	0.015	98.95 ± 8.01	0.210 ± 0.042
	0.03	148.04 ± 0.00	0.066 ± 0.022
	0.04	142.28 ± 8.15	0.071 ± 0.058
	0.05	126.17 ± 10.21	0.039 ± 0.049
	0.06	113.35 ± 10.87	0.098 ± 0.073
Speed(rpm)	200	107.30 ± 8.68	0.147 ± 0.030
	400	126.17 ± 10.21	0.039 ± 0.049
	600	116.36 ± 9.42	0.054 ± 0.042
	800	85.87 ±34.66	0.247 ± 0.083
	1000	111.59 ± 6.39	0.171 ± 0.083
Acetonitrile (%)	2.5	——	——
	5	126.17 ± 10.21	0.039 ± 0.049
	10	119.37 ± 5.66	0.128 ± 0.013
	20	104.53 ± 10.02	0.152 ± 0.016
	50	9.32 ± 4.83	0.230 ± 0.039

## Data Availability

Not applicable.
